# Adjuvant Therapy for Patients with a Tumor-Positive Resection Margin After Neoadjuvant Chemoradiotherapy and Esophagectomy

**DOI:** 10.1245/s10434-024-14912-x

**Published:** 2024-01-20

**Authors:** C. J. van der Zijden, P. C. van der Sluis, B. Mostert, J. J. M. E. Nuyttens, V. M. C. W. Spaander, R. Valkema, J. P. Ruurda, B. P. L. Wijnhoven, S. M. Lagarde

**Affiliations:** 1 Department of Surgery, Erasmus MC Cancer Institute, Erasmus University Medical Center, Rotterdam, The Netherlands; 2 Department of Medical Oncology, Erasmus MC Cancer Institute, Rotterdam, The Netherlands; 3 Department of Radiation Oncology, Erasmus MC Cancer Institute, Rotterdam, The Netherlands; 4 Department of Gastroenterology and Hepatology, Erasmus University Medical Center, Rotterdam, The Netherlands; 5 Department of Nuclear Medicine, Erasmus University Medical Center, Rotterdam, The Netherlands; 6 Department of Surgery, University Medical Center Utrecht, Utrecht, The Netherlands

**Keywords:** Esophageal cancer, Neoadjuvant chemoradiotherapy, Tumor-positive resection margin, Adjuvant therapy

## Abstract

**Background:**

Approximately 4–9% of patients have a tumor-positive resection margin after neoadjuvant chemoradiotherapy (nCRT) and esophagectomy. Although it is associated with decreased survival, Western guidelines do not recommend adjuvant treatment.

**Objective:**

The aim of this study was to assess the proportion of patients who received adjuvant therapy, and to evaluate overall survival (OS) after esophagectomy in patients with a tumor-positive resection margin.

**Methods:**

Patients diagnosed with resectable (cT2-4a/cTxN0-3/NxM0) esophageal cancer between 2015 and 2022, and treated with nCRT followed by irradical esophagectomy, were selected from the Netherlands Cancer Registry. The primary outcome was the proportion of patients with a tumor-positive resection margin who started adjuvant treatment ≤16 weeks after esophagectomy, including chemotherapy/radiotherapy, immunotherapy, or targeted therapy. OS was calculated from the date of surgery until the date of death or last day of follow-up.

**Results:**

Overall, 376 patients were included in our study, of whom 357 were treated with nCRT. Of these 357 patients, 98.3% had a microscopically irradical resection and 1.7% had a macroscopically irradical resection. Approximately 72.3% of tumors showed a partial response (Mandard 2–3) and 11.8% showed little/no pathological response (Mandard 4–5) to nCRT. One of 357 patients underwent adjuvant chemoradiotherapy and 39 patients (61%) underwent adjuvant immunotherapy (nivolumab). The median and 5-year OS rate of all patients was 16.4 months (95% confidence interval 13.1–19.8) and 21%, respectively.

**Conclusion:**

Real-world population-level data showed that no patients with a tumor-positive resection margin underwent adjuvant therapy following nCRT and esophagectomy prior to 2021. Interestingly, 61% of patients were treated with adjuvant nivolumab in 2021–2022. OS after irradical esophagectomy is poor and long-term data will explore the added value of nivolumab.

Since the CROSS trial showed improved survival of patients with locally advanced esophageal cancer after neoadjuvant chemoradiotherapy (nCRT) and esophagectomy, trimodality therapy has become standard of care in The Netherlands.^1–3^ Despite nCRT decreasing irradicality, a tumor-positive resection margin has still been reported in 4–9% of patients nationwide.^3^ A recent study showed that tumor length, cT4 stage, and performing an Ivor Lewis esophagectomy are risk factors for tumor-positive resection margins after nCRT and esophagectomy.^4^ Being aware of these risk factors could make it possible to improve radical resection rates through better selection of patients and through optimizing the right surgical approach.

As of 2022, this therapy has been expanded with adjuvant nivolumab for patients who have residual disease (ypT+N0 or ypT0N+) in the resection specimen.^5^ No recommendations for adjuvant therapy have been made in current guidelines to date.^6–8^ Nevertheless, opinions differ on the sense and non-sense of adjuvant therapy after esophagectomy with tumor-positive resection margins, and therefore remains a matter of debate. In accordance with current guidelines and based on the inclusion criteria of the Checkmate 577 trial, the presence of a tumor-negative resection margin is required for adjuvant treatment with nivolumab.^5^ However, the presence of tumor-positive resection margins is associated with worse survival due to early occurrence of local recurrence or distant metastases.^9^ Although nivolumab has only been registered after nCRT and radical esophagectomy, it could be hypothesized that patients with a tumor-positive resection margin may benefit from adjuvant nivolumab.

Thus, the aim of this study was to gain insight into the proportion of patients who received adjuvant therapy after esophagectomy with a tumor-positive resection margin.

## Materials and Methods

### Study Design and Patients

Patients diagnosed with resectable (cT2-4a/cTx, N0-3/Nx, M0) esophageal or junctional cancer between 2015 and 2022 were selected from the Netherlands’ Cancer Registry (NCR), a nationwide population-based cancer registry that covers the entire Dutch population of more than 17 million people. The NCR is based on the notification of all newly diagnosed malignancies in The Netherlands by the national automated pathology archive (PALGA). Specially trained employees from the NCR routinely extract additional information on diagnosis, tumor stage, and treatment from the medical records. Only patients who were treated with nCRT according to the CROSS regimen followed by esophagectomy with a tumor-positive resection margin were included.

### Neoadjuvant Chemoradiotherapy

The regimen consists of five weekly cycles of intravenous carboplatin at an area under the curve (AUC) of 2 mg/mL/min and intravenous paclitaxel at a dose 50 mg/m^2^ on the first day of each week, with concurrent radiotherapy of 41.4 Gy administered in 23 fractions of 1.8 Gy for 5 days per week, starting on the first day of each cycle of chemotherapy.^3^

### Surgery

Surgery was performed using an open, laparoscopic, or robot-assisted approach, while esophagectomy was performed using either a transhiatal (i.e., Orringer) with gastric conduit reconstruction and cervical anastomosis approach, transthoracic (i.e., Ivor Lewis or McKeown) with gastric conduit reconstruction and cervical or intrathoracic anastomosis approach, or cervical (i.e., minimally invasive cervical esophagectomy [MICE]) approach, which combines a laparoscopic transhiatal and single-port transcervical mediastinal dissection. As an alternative, patients with distal/junctional tumors could also undergo an extended (minimally invasive) total gastrectomy with gastrointestinal reconstruction.

### Resection Specimen

Resected tumors were staged according to the Union for International Cancer Control/American Joint Committee on Cancer (UICC/AJCC) TNM staging manual.^10,11^ Radical resection (R0) was defined as no contact between tumor and surgical margin (clearance of 0.0 cm), and in the case of microscopically irradical resection (R1), there is contact between tumor and surgical margin (i.e., resection margin ≤1 mm).^12^ A tumor-positive resection margin could be located either proximal, distal, or circumferential. A macroscopically irradical resection (R2) is defined as visible residual tumor that is left behind during surgery and cannot be resected because of ingrowth in surrounding organs or tissues.

### Outcomes

The primary outcome was the proportion of patients who were treated with adjuvant therapy ≤16 weeks after esophagectomy, including chemotherapy, immunotherapy, or targeted therapy. The reason for this 16-week cut-off is that therapy must have been started within this postoperative period to consider it as adjuvant therapy. Secondary outcomes were the proportion of patients treated with adjuvant nivolumab from the moment it was reimbursed in the Dutch healthcare system (i.e., January 2022), and overall survival (OS).

### Statistical Analyses

Patient and tumor characteristics were analyzed using descriptive statistics and were presented as mean, median with interquartile range (IQR), or frequencies (%). The proportion of patients treated with adjuvant therapy was calculated relative to all patients who underwent nCRT and had an irradical esophagectomy, while the proportion of patients treated with adjuvant nivolumab was calculated relative to one-third of patients diagnosed in 2021 (as this group of patients could have been eligible for nivolumab after a radical esophagectomy) and all patients diagnosed in 2022. Survival was reported in months and was calculated from the date of esophagectomy until the date of death or last day of follow-up, using the Kaplan–Meier method. Statistical analysis was performed using R version 4.0.0 (R: A language and environment for statistical computing; R Foundation for Statistical Computing, Vienna, Austria).

## Results

**Fig. 1:**
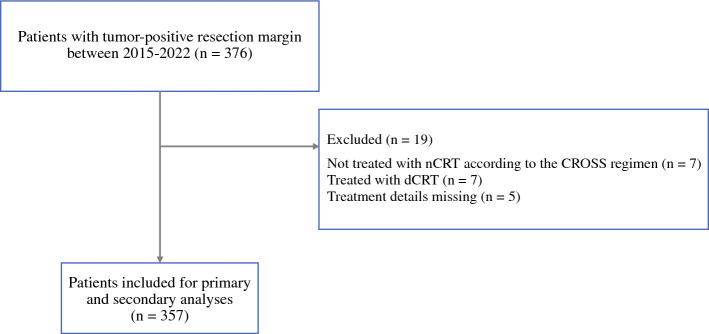
Patient selection process. *nCRT* neoadjuvant chemoradiotherapy, *dCRT* definitive chemoradiotherapy

In total, 4734 patients underwent nCRT and esophagectomy between 2015 and 2022; 376 (7.5%) patients had tumor-positive resection margins and were therefore included in this study, of whom seven patients were not treated with nCRT according to the CROSS regimen, another seven patients were treated with definitive chemoradiotherapy, and for five patients, details about neoadjuvant therapy were missing, leaving 357 patients for analyses of primary and secondary outcomes (Fig. 1). Baseline characteristics are presented in Table 1 and details about surgery are shown in Table 2.

**Table 1:** Patient and tumor characteristics

Characteristics	All patients [*n* = 357] (%)
Male sex	277 (77.6)
Age, years (median [IQR])	66 [60–72]
WHO performance status	
0–1	321 (89.9)
2	9 (2.5)
3	1 (0.3)
Missing	26 (7.3)
Tumor type	
Adenocarcinoma	304 (85.2)
Squamous cell carcinoma	47 (13.2)
Other	6 (1.7)
Tumor location	
Middle esophagus	38 (10.6)
Distal esophagus/EGJ/cardia	319 (89.4)
Tumor differentiation grade	
Well differentiated (G1)	–
Moderately differentiated (G2)	137 (38.4)
Poorly differentiated (G3)	187 (52.4)
Undifferentiated (G4)	1 (0.3)
Missing	32 (9)
Clinical T category	
cTx	5 (1.4)
cT1	–
cT2	72 (20.2)
cT3	269 (75.4)
cT4a	11 (3.1)
Clinical N category	
cN0	145 (40.6)
cN1	131 (36.7)
cN2	69 (19.3)
cN3	10 (2.8)
Missing	2 (0.6)

*EGJ* esophagogastric junction

**Table 2:** Surgical characteristics

Characteristics	All patients [*n* = 357] (%)
Surgical technique	
Open resection	26 (7.3)
Minimally invasive	328 (91.9)
Missing	3 (0.8)
Surgical approach	
Transhiatal esophagectomy	31 (8.7)
Transthoracic esophagectomy	321 (89.9)
MICE	2 (0.6)
Total gastrectomy	3 (0.8)
Radicality of resection	
Microscopically irradical (R1)	351 (98.3)
Macroscopically irradical (R2)	6 (1.7)
Tumor regression grade	
Subtotal pathological response	44 (12.3)
Partial pathological response	258 (72.3)
No pathological response	42 (11.8)
Missing	1 (0.3)
Pathological T category	
ypTx	3 (0.8)
ypT1b	7 (2)
ypT2	26 (7.3)
ypT3	297 (83.2)
ypT4a	16 (4.5)
ypT4b	8 (2.2)
Pathological N category	
ypN0	119 (33.3)
ypN1	101 (28.3)
ypN2	70 (19.6)
ypN3	60 (16.8)
Missing	7 (2)
Pathological M category	
ypM1	13 (3.6)

*MICE* minimally invasive cervical esophagectomy

### Resection Specimen

Most patients had a microscopically irradical (R1) resection (98.3%) and six patients had a macroscopically irradical (R2) resection (1.7%). For most patients, data regarding the location of tumor-positive resection margins (i.e., proximal, distal or circumferential) were missing.

### Adjuvant Therapy

Approximately 40 patients (11.2%) underwent adjuvant therapy ≤16 weeks after irradical resection, all during the years 2021 and 2022. With the exception of one patient who was treated with chemoradiation, all remaining patients were treated with nivolumab. Thirty-nine patients (60.9%) were treated with adjuvant nivolumab after it was reimbursed for non-pathological responders, out of a total of 64 patients with an irradical esophagectomy. The median time between resection and the start of adjuvant therapy was 10.4 weeks (IQR 6.9–13.9).

### Survival

The median OS of all patients who underwent irradical resection was 16.4 months (95% confidence interval [CI] 13.1–19.8), with corresponding 2-, 3-, and 5-year OS rates of 37.5% (95% CI 32.2–43.8%), 27.1% (95% CI 22.0–33.4%), and 21% (95% CI 15.9–27.6), respectively. Survival for patients treated with adjuvant therapy could not be calculated due to the limited follow-up.

## Discussion

These real-world population-level data showed that the majority of patients with locally advanced esophageal cancer did not undergo adjuvant therapy following nCRT and esophagectomy with a tumor-positive resection margin. Only 11% of patients underwent adjuvant therapy and nearly all of those patients were treated with nivolumab. Our findings shed light on the frequency that adjuvant therapy was administered for this specific patient population in current clinical practice.

Adjuvant therapy in patients with a tumor-positive resection margin is controversial. The NCCN guideline recommends chemoradiotherapy or palliative management, while the European Society for Medical Oncology (ESMO), American Society of Clinical Oncology (ASCO), and Asian guidelines (Japan Endocrine Society [JES]) recommend adjuvant therapy after irradical esophagectomy; this remains an area of ongoing research.^6–8,13^ Some studies reported that adjuvant therapy (either chemo[radio]therapy or radiotherapy) improves recurrence-free survival and OS, but possibly only in a selected group of patients who remain to be identified.^14,15^ The decision to recommend adjuvant therapy should be discussed in a multidisciplinary team and individualized, taking into account factors such as the patient’s performance status, tumor characteristics, the location of tumor-positive resection margins, and potential treatment-related toxicities.

Immunotherapy for esophageal cancer has been a topic of significant interest and analysis over the past years. Based on the results of the Checkmate 649, ATTRACTION-3, and Checkmate 577 trials, nivolumab has been approved for clinical use by the US Food and Drug Administration (FDA) and the European Medicines Agency (EMA) as first- and second-line palliative treatment, and as adjuvant therapy in patients with residual tumor in the resection specimen following nCRT and esophagectomy.^5,16,17^ However, patients with a tumor-positive resection margin were not included in the Checkmate 577 trial and therefore nivolumab is not recommended in these patients.^18^ The present study shows that 39 patients with a tumor-positive resection margin were treated with adjuvant nivolumab. One can argue that clinicians who opted for adjuvant nivolumab were potentially driven by the need to mitigate the increased risk of recurrence associated with a tumor-positive resection margin. Patients who are treated with systemic therapy outside the registration criteria are exposed to adverse effects and the burden of hospital visits and interventions, while the benefit is unclear. Furthermore, we should consider the cost effectiveness of each treatment. In The Netherlands, nivolumab costs €5298 per 28-day treatment cycle, bringing the total costs of 1 year of treatment, not taking into account costs for outpatient clinic visits, blood draws and CT scans, to €68,870 per patient.^19^ As the effectiveness of nivolumab in patients with a tumor-positive resection margin has not been studied, we feel it inappropriate to prescribe an expensive treatment such as nivolumab until new studies showing a benefit are available. We would therefore recommend performing a phase II intervention study to explore the efficacy in terms of OS and potential adverse effects, as well as the cost effectiveness of adjuvant nivolumab, in those patients and to cease the off-label use of nivolumab until supporting evidence is available.

The secondary outcome, OS, was comparable with previous studies reporting on patients with irradical esophagectomy after neoadjuvant chemo(radio)therapy, with a median OS of 16.4 months and corresponding 5-year OS rate of 21%.^9^ It is important to realize that this concerns a selection of patients, as radical esophagectomy was achieved in 95% of patients after CROSS chemoradiotherapy.^20^ However, patients with irradical esophagectomy have a 5-year survival loss of more than 30 months and 26% compared with patients treated with nCRT and esophagectomy (radical or not), which provides room for improvement.^2^ The relatively low survival rates highlight the challenges in managing this aggressive disease. Future research efforts should focus on refining surgical techniques, exploring the role of adjuvant therapies, developing predictive biomarkers, and establishing evidence-based guidelines.

This study benefits from the large dataset derived from a national registry to enable a comprehensive evaluation of clinical practice within a Western population. However, it is crucial to acknowledge that the findings of this study may not be universally applicable since the clinical use of adjuvant therapy can vary among hospitals, regions, and even countries. Instead, the findings serve as a valuable prompt for healthcare professionals in other hospitals or countries to evaluate the use of adjuvant therapy following esophagectomy in patients with a tumor-positive resection margin. The small number of patients who receive adjuvant therapy limits our ability to draw definitive conclusions about its efficacy in this specific context. Furthermore, the lack of detailed information on the location of tumor-positive resection margins (i.e., proximal, distal, or circumferential) hinders our understanding of the impact of margin location on the decision to administer adjuvant therapy. Depending on the location, the risk of cancer recurrence and the potential benefits of adjuvant therapy might vary.

## Conclusion

Our study provides insight into the utilization of adjuvant therapy in patients with a tumor-positive resection margin following nCRT and esophagectomy for locally advanced esophageal cancer in clinical practice. Despite the absence of guidelines supporting adjuvant therapy with nivolumab, a subset of patients received immunotherapy within 16 weeks from resection. Further research is warranted to determine the potential benefit of adjuvant therapy in patients with a tumor-positive resection margin and to identify optimal treatment strategies to improve outcomes in this challenging subset of esophageal cancer patients.

## References

[1] Eyck BM, van Lanschot JJB, Hulshof M, van der Wilk BJ, Shapiro J, van Hagen P (2021). Ten-year outcome of neoadjuvant chemoradiotherapy plus surgery for esophageal cancer: the randomized controlled CROSS trial. J Clin Oncol..

[2] Shapiro J, van Lanschot JJB, Hulshof M, van Hagen P, van Berge Henegouwen MI, Wijnhoven BPL (2015). Neoadjuvant chemoradiotherapy plus surgery versus surgery alone for oesophageal or junctional cancer (CROSS): long-term results of a randomised controlled trial. Lancet Oncol..

[3] van Hagen P, Hulshof MC, van Lanschot JJ, Steyerberg EW, van Berge Henegouwen MI, Wijnhoven BP (2012). Preoperative chemoradiotherapy for esophageal or junctional cancer. N Engl J Med..

[4] Defize IL, Goense L, Borggreve AS, Mook S, Meijer GJ, Ruurda JP (2023). Risk factors for tumor positive resection margins after neoadjuvant chemoradiotherapy for esophageal cancer: results from the dutch upper GI cancer audit: a nationwide population-based study. Ann Surg..

[5] Kelly RJ, Ajani JA, Kuzdzal J, Zander T, Van Cutsem E, Piessen G (2021). Adjuvant nivolumab in resected esophageal or gastroesophageal junction cancer. N Engl J Med..

[6] Manish AS, Erin BK, Daniel VC, Dana CD, Karyn AG, Narinder KM (2020). Treatment of locally advanced esophageal carcinoma: ASCO guideline. J Clin Oncol..

[7] Lordick F, Mariette C, Haustermans K, Obermannová R, Arnold D, on behalf of the ESMO Guidelines Committee. Oesophageal cancer: ESMO Clinical Practice Guidelines for diagnosis, treatment and follow-up. Ann Oncol. 2016; 27 Suppl 5:v50-v7. 10.1093/annonc/mdw329 27664261

[8] Kitagawa Y, Uno T, Oyama T, Kato K, Kato H, Kawakubo H (2019). Esophageal cancer practice guidelines 2017 edited by the Japan Esophageal Society: part 1. Esophagus..

[9] Markar SR, Gronnier C, Duhamel A, Pasquer A, Théreaux J, Chalret du Rieu M (2016). Significance of microscopically incomplete resection margin after esophagectomy for esophageal cancer. Ann Surg..

[10] Rice TW, Patil DT, Blackstone EH (2017). 8th edition AJCC/UICC staging of cancers of the esophagus and esophagogastric junction: application to clinical practice. Ann Cardiothorac Surg..

[11] Rice TW, Blackstone EH, Rusch VW (2010). 7th edition of the AJCC cancer staging manual: esophagus and esophagogastric junction. Ann Surg Oncol..

[12] CAP. Protocol for the examination of specimens from patients with carcinoma of the esophagus, 2017, June. Available at: https://documents.cap.org/protocols/cp-esophagus-17protocol-4000.pdf. Accessed 23 Mar 2023.

[13] Ajani JA, D’Amico TA, Bentrem DJ, Chao J, Corvera C, Das P (2019). Esophageal and esophagogastric junction cancers, version 2.2019, NCCN clinical practice guidelines in oncology. J Natl Compr Canc Netw..

[14] Raman V, Jawitz OK, Voigt SL, Yang CJ, D'Amico TA, Harpole DH (2020). The role of adjuvant therapy in patients with margin-positive (R1) esophagectomy: a national analysis. J Surg Res..

[15] Bott RK, Beckmann K, Zylstra J, Wilkinson MJ, Knight WRC, Baker CR (2020). Adjuvant therapy following oesophagectomy for adenocarcinoma in patients with a positive resection margin. Br J Surg..

[16] Janjigian YY, Shitara K, Moehler M, Garrido M, Salman P, Shen L (2021). First-line nivolumab plus chemotherapy versus chemotherapy alone for advanced gastric, gastro-oesophageal junction, and oesophageal adenocarcinoma (CheckMate 649): a randomised, open-label, phase 3 trial. Lancet..

[17] Kato K, Cho BC, Takahashi M, Okada M, Lin CY, Chin K (2019). Nivolumab versus chemotherapy in patients with advanced oesophageal squamous cell carcinoma refractory or intolerant to previous chemotherapy (ATTRACTION-3): a multicentre, randomised, open-label, phase 3 trial. Lancet Oncol..

[18] Richtlijnendatabase Oesofaguscarcinoom (2010). Federatie Medisch Specialisten. Available at: https://richtlijnendatabase.nl/richtlijn/oesofaguscarcinoom/oesofaguscarcinoom_-_startpagina.html. Accessed 14 Jun 2023.

[19] NVMO-commissie BOM (2021). Adjuvant nivolumab bij het oesofaguscarcinoom of carcinoom van de gastro-oesofageale overgang na neoadjuvante chemoradiatie en resectie. Available at: https://www.nvmo.org/bom/adjuvant-nivolumab-bij-het-oesofaguscarcinoom-of-carcinoom-van-de-gastro-oesofageale-overgang-na-neoadjuvante-chemoradiatie-en-resectie/. Assessed 14 Jun 2023.

[20] John VR, Shaun RP, Brian ON, Maeve Aine L, Lene B, Thomas C (2021). Neo-AEGIS (neoadjuvant trial in adenocarcinoma of the esophagus and esophago-gastric junction international study): preliminary results of phase III RCT of CROSS versus perioperative chemotherapy (modified MAGIC or FLOT protocol). J Clin Oncol..

